# A 62-Year-Old Male Patient With Spinal Lesions Following Fingolimod Discontinuation in the Setting of Disseminated Shingles

**DOI:** 10.7759/cureus.84587

**Published:** 2025-05-21

**Authors:** Michelle Y Ko, Eric Williamson

**Affiliations:** 1 Medicine, University of California Los Angeles David Geffen School of Medicine, Los Angeles, USA; 2 Neurology, University of California Los Angeles David Geffen School of Medicine, Los Angeles, USA

**Keywords:** covid-19, fingolimod, ms (multiple sclerosis), ocrelizumab, rebound, varicella-zoster virus

## Abstract

The association between the discontinuation of therapies and “rebound” multiple sclerosis (MS) activity has been frequently reported, yet is incompletely understood. We report an older male with previously well-controlled MS who was taken off his decade-long fingolimod treatment due to disseminated zoster infection, who subsequently experienced two severe MS exacerbations within six weeks. Though clinicians should consider possible confounding influences from infections such as varicella-zoster virus (VZV) and COVID-19, this case raised the most concern for suspicion of early fingolimod-associated rebound.

## Introduction

Multiple sclerosis (MS) is a potentially debilitating disease characterized by inflammation resulting in lesions to the central nervous system (CNS). Recent studies show that the prevalence of MS is rising, with about 2.8 million people living with MS worldwide as of 2020 [1]. At the same time, disease-modifying therapies (DMTs) for MS have been evolving, with current drug options including interferons, glatiramer acetate, sphingosine 1-phosphate receptor modulators, cladribine, fumarates, teriflunomide, and monoclonal antibodies [2].

In 2004, natalizumab was one of the first high-efficacy DMTs approved for relapsing-remitting MS (RRMS) in adults. Natalizumab, an infusion, is a humanized IgG4 monoclonal antibody that acts as a selective adhesion molecule antagonist, thought to prevent the migration of leukocytes across blood vessel membranes, such as the blood-brain barrier (BBB) [3]. Despite natalizumab’s efficacy in treating RRMS, its association with progressive multifocal leukoencephalopathy (PML) has limited its use and made its discontinuation necessary in some patients [3]. Over the years, the association between the discontinuation of natalizumab and “rebound” MS activity has been frequently reported, well studied, and largely accepted, hypothesized to be the result of reactivation of immune cells and cytokines flooding into peripheral blood and past BBB [4]. Rebound associated with fingolimod, the first oral therapy approved for MS in 2010, is less well-established. Fingolimod is a sphingosine-1-phosphate receptor modulator, which is thought to be a primarily T cell modulator, sequestering T cells into lymph nodes and affecting T cell function, though it also has been shown to affect B cell function [5]. Similar to natalizumab, fingolimod's "antitrafficking" properties of reducing CNS entry of autoreactive lymphocytes may help explain potential rebound phenomena when the drug is discontinued. Although the foundational FREEDOMS I and II studies found no difference in high disease activity after the discontinuation of fingolimod vs. placebo drug [6], several case reports and retrospective studies since have documented evidence of fingolimod rebound, with studies estimating that 10% of these patients experience this phenomenon [7,8].

This case report was presented as a poster at the 2023 Americas Committee for Treatment and Research in Multiple Sclerosis (ACTRIMS) Forum and as a podium presentation at the 2023 Paralyzed Veterans of America (PVA) Healthcare Summit and Expo.

## Case presentation

A 62-year-old male patient with MS previously well-controlled on fingolimod for the past nine years presented with disseminated varicella-zoster virus (VZV). Of note, the patient had childhood chickenpox, had no prior episodes of shingles, and was just vaccinated against VZV with the zoster recombinant vaccine the year prior. The patient was treated with valacyclovir, and fingolimod was discontinued due to the known association between fingolimod and the reactivation of VZV. The patient was instructed to follow up outpatient for a trial of five days of 1 g of methylprednisolone, followed by the initiation of a new DMT in the form of ocrelizumab. However, the patient was unable to be contacted and lost to follow-up.

**Figure 1:**
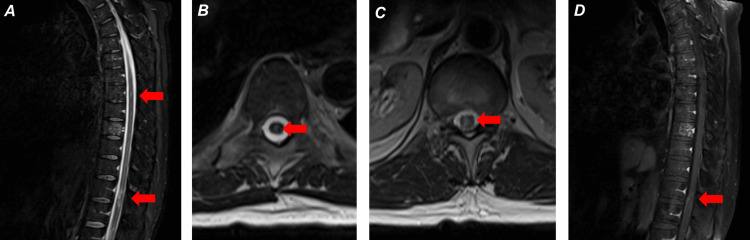
First post-fingolimod MS exacerbation: (A) (sagittal thoracic spine) T2/STIR signal hyperintense lesions at T7 (top red arrow) and T12-L1 (bottom red arrow), (B) (axial thoracic spine) T2/STIR signal hyperintense lesion at T7 (red arrow), (C) (axial thoracic spine) T2/STIR signal hyperintense lesion at T12-L1 (red arrow), and (D) (sagittal thoracic spine) T1 contrast enhancement at the T12-L1 lesion (red arrow). No enhancement at the T7 lesion (not pictured). MS: multiple sclerosis; T2/STIR: T2-weighted short-tau inversion recovery

**Figure 2:**
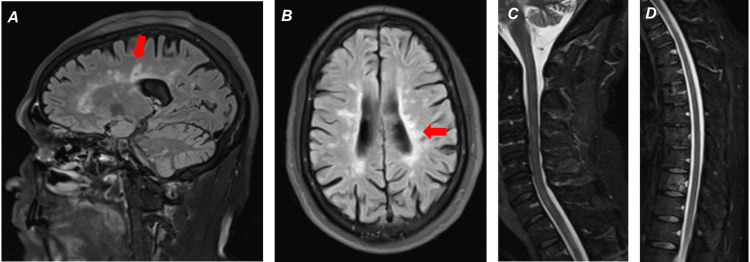
10 months prior: T2 imaging demonstrating (A) (sagittal brain) Dawson’s fingers (red arrow), (B) (axial brain) hyperintense periventricular lesions (red arrow), (C) (sagittal cervical spinal cord), and (D) (sagittal thoracic spinal cord) demonstrating the absence of lesions that developed below.

A month later, the patient presented to the emergency department (ED) with worsened numbness, tingling, and weakness that had progressed from the bilateral lower extremities to the pelvis and worsened incontinence. The patient also endorsed an increasing number of falls and drowsiness since his last admission. The neurological exam was notable for increased muscle tone, decreased sensation, and decreased strength (approximately 4/5) of the bilateral lower extremities, with the right worse than the left. Lower extremity deep tendon reflexes were brisk (2+ to 3+) with sustained clonus of the left ankle. The patient was alert and oriented to person, time, place, and purpose. MRI of the brain and cervical spine showed no significant changes from prior or evidence of active demyelinating disease or inflammation. MRI of the thoracic spine revealed new lesions at T12-L1 and T7 (Figures 1A-1D), which were not present 10 months prior (Figures 2A-2D). These were his first new lesions since at least eight years prior. The patient received five days of methylprednisolone infusion with some recovery. Fourteen days after his ED presentation, the patient received his first course of ocrelizumab infusion.

**Figure 3:**
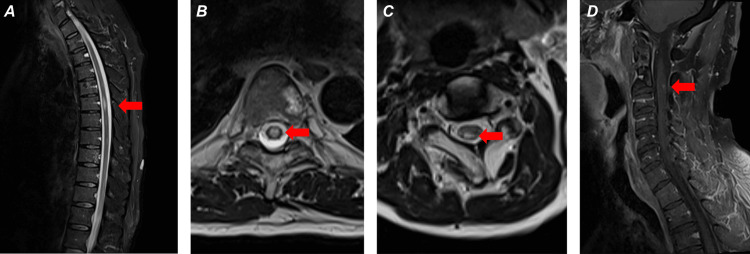
Second post-fingolimod MS exacerbation: (A) (sagittal thoracic spine); (B) (axial thoracic spine) demonstrating T2 prolongation at T6 (red arrow), encompassing almost the entire cord; (C) (axial cervical spine) T2 prolongation at C2-C3 (red arrow), within the posterior predominantly right hemicord; (D) (sagittal cervical spine) T1 contrast enhancement at the C2-C3 lesion (red arrow). Enhancement at the T6 lesion present (but is not pictured). MS: multiple sclerosis

Within five days of receiving ocrelizumab, the patient experienced worsening lower extremity numbness, weakness, falls, and somnolence. A neurological exam revealed decreased sensation and decreased strength (3 to 4/5) of the bilateral lower extremities with largely unchanged deep tendon reflexes. The patient was sleepy but arousable to voice and responded appropriately once awakened and oriented to person, time, place, and purpose. The patient had bradypnea, with a respiratory rate of eight breaths per minute, and hypercarbia (Tables 1, 2). However, the patient continued to have good inspiratory effort (serial negative inspiratory force (NIF) of -40), SaO_2_ of 100% on 2 L of nasal cannula, and no apparent distress. The patient was afebrile, and the chest x-ray and urine analysis were unremarkable. His serum IgG for varicella zoster was positive (Table 2), though unfortunately VZV DNA and IgM levels were not measured. He was incidentally found to be asymptomatically COVID-positive. CT of the head was negative for intracranial bleeding. MRI revealed new lesions at C2-C3 and T6 (Figures 3A-3D). MRI of the brain was concurrently performed and confirmed stable. The patient was treated with three days of remdesivir and five days of methylprednisolone, quickly demonstrating marked improvement in wakefulness and lower extremity strength and sensation.

**Table 1:** Patient's vital signs during his second exacerbation. F: Fahrenheit; C: Celsius; L: liters; NC: nasal cannula

Vital sign	Value
Temperature	99°F (37.2°C)
Pulse	71 beats per minute
Blood pressure	123/89 mmHg
Respiration rate	8-16 breaths per minute
Oxygen saturation	100% on 2 L NC

**Table 2:** Corresponding lab values for infectious workup, basic metabolic panel, liver enzyme/function tests, and venous blood gas. RT-PCR: reverse transcription polymerase chain reaction; ALT: alanine aminotransferase; AST: aspartate aminotransferase *Reference value for random, non-fasting glucose.

	Patient value	Reference range
White blood cell count	3.78 K/μL	4.5-11 K/μL
Varicella-zoster IgG	>4,000 AI (antibody index)	<135 AI
COVID-19 (RT-PCR)	Positive	Negative
Sodium	137 mmol/L	136-146 mEq/L
Potassium	4.1 mmol/L	4.5-5.0 mEq/L
Creatinine	1.19 mg/dL	0.6-1.2 mg/dL
Glucose	81 mg/dL	<140 mg/dL*
ALT	25 U/L	10-40 U/L
AST	20 U/L	12-38 U/L
Alkaline phosphatase	65 U/L	25-100 U/L
Total bilirubin	0.5 mg/dL	0.1-1.0 mg/dL
Venous blood gas pH	7.32	7.35-7.45
Venous blood gas pCO_2_	55 mmHg	33-45 mmHg

**Figure 4:**
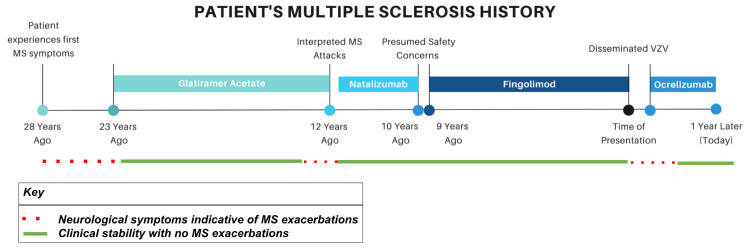
Timeline of the patient’s multiple sclerosis (MS) history, including his disease-modifying therapy regimen since diagnosis, his clinical stability on those medications, and this case presentation. VZV: varicella-zoster virus

The patient’s past history with MS is as follows (Figure 4): The patient reported initial symptoms 28 years ago when he became vertiginous on upward gaze. The following year, he was officially diagnosed with MS given his neurological episodes separated by time and space, confirmatory MRI findings, and lumbar puncture (LP) revealing IgG synthesis of 6.39 and 4 oligoclonal bands (Expanded Disability Status Scale (EDSS): 1-2). The patient continued having perceived MS exacerbations until starting glatiramer acetate from 23 to 12 years ago (EDSS: 4) but was switched to another medication due to interpreted attacks, namely, an increasing number of falls (EDSS: 5). He was then transitioned to natalizumab from 12 to 10 years ago, and despite stability, it was stopped presumably due to safety concerns (EDSS: 5.5). Though the exact reason for discontinuation was not documented in the chart, it appears the patient was stopped on natalizumab after patient discussion due to the increased risk for PML associated with an increasing number of natalizumab infusions. The patient had no documented history of PML and no documentation of any testing for John Cunningham virus (JCV).

The patient was then started on fingolimod for the following nine years (EDSS: 6-6.5), during which he endorsed stability and denied exacerbations, which was confirmed by yearly MRI brain scans that confirmed no gross new plaques. MRI spine scans were limited by the fact that they were not being performed yearly until one year prior to our case presentation. The patient was stable while on fingolimod until discontinuation of the drug due to disseminated VZV, when he experienced the MS exacerbations described in this case. After continuing ocrelizumab for one year, the patient retained clinical stability (EDSS: 6.5).

## Discussion

A number of differential diagnoses were considered for this patient's increasing lower extremity weakness and somnolence. Infectious etiology, such as Guillain-Barre or Lyme disease, was less likely given the patient's lack of infectious contacts and symptoms, as the patient was afebrile, without a leukocytosis, and with a non-contributory urine analysis and chest x-ray. A CT scan of the head was obtained given the patient's history of falls but was negative for intracranial bleeding. A basic metabolic panel was within normal limits, making metabolic derangements such as uremia or lactic acidosis less likely as well. MRI revealed new lesions along the spinal cord at C2-C3 and T6 suggestive of new and active demyelinating lesions, which could have explained the patient's bradypnea, given C3's involvement in diaphragm innervation, which could have subsequently contributed to the patient's somnolence. Ultimately, the timing of the onset of symptoms, evidence of worsening MS symptoms, and MRI evidence of new demyelinating lesions strongly suggest MS exacerbation due to rebound associated with the discontinuation of fingolimod.

Several studies found that the main reason for discontinuing fingolimod in their patients was therapeutic failure and inefficacy [9]. Other reasons included pregnancy planning, increased liver function enzymes, and severe VZV infection associated with fingolimod use [8-10]. Discontinuation of fingolimod was felt to be justified, given its known association with severe herpetic infections and the patient’s disseminated zoster, especially given long-standing disease quiescence.

Fingolimod rebound remains difficult to confirm partially because it has been inconsistently defined. While rebound generally refers to “severe relapse” after withdrawal of DMT, whether that activity is worse compared to a patient’s baseline disease activity pattern prior to treatment initiation or refers to a return to the earlier state of disease is in question [5]. Further complicating definitions of the phenomena are inconsistencies in the time frame within which disease activity would qualify as “rebound activity,” with some publications referring to activity within three months [8] and other studies referring to activity within six months [11]. Additionally, different studies have defined “severe relapse” using various measurements, such as the presence of new severe neurological symptoms, relapses associated with hospitalizations or incomplete recovery, increases in Kurtzke EDSS scores, or a specific minimum number of new enhancing MRI lesions [5,12]. Our patient seems to meet the criteria for rebound in various ways, given the increased frequency of these two closely timed relapses with confirmed MRI imaging only about four weeks apart from one another and within weeks of his fingolimod discontinuation. Prior to beginning his MS treatment in 1998, the patient had MS exacerbations separated by years. Additionally, these exacerbations were severe given that the first resulted in incomplete recovery according to the patient, the second required hospitalization, and each was associated with severe symptoms with multiple new, enhancing MRI lesions.

A recently published case series reported three distinct MRI brain presentations of fingolimod rebound: first, a pattern more consistent with classic MS; second, tumefactive lesions, defined as a solitary demyelinating lesion greater than 2 cm; and third, a punctuated pattern of innumerable small T2 and gadolinium-enhancing lesions [5,13]. Fewer case series have focused on the characteristics of spinal lesions, though several studies report the presence of several gadolinium-enhancing lesions from rebound in the thoracic and cervical regions [14]. For our patient, both of his exacerbations were associated with isolated disease reactivation in the cervical and thoracic spinal regions, with brain MRIs remaining stable since the last imaging taken last year, which could represent a unique, spinal-dominant variant of fingolimod rebound.

Different studies have reported different times for patients to present with severe relapse after cessation of fingolimod treatment. Barboza et al. report a median of 50 days, Berger et al. report a range of 2-4 months (mean 2.5 months), Hatcher et al. report a range of 4-16 weeks (mean 7.6 weeks), and Uygunoglu et al. report a range of 2-3 months (median 3 months) [5,9,10,12,15]. Our patient experienced his first severe relapse at one month and a second severe relapse at six weeks post-discontinuation of fingolimod. The shorter time for fingolimod relapse, in comparison, for example, to natalizumab, which has been reported to have rebound at an average of 4.5 months after discontinuation [4], is consistent with fingolimod’s relatively short half-life of 6-9 days [16], compared to natalizumab’s half-life of 16 ± 4 days [3].

The mechanism of action for fingolimod rebound is still not fully understood. One current hypothesis proposes that discontinuing fingolimod allows lymphocytes that had previously been kept at bay in lymph nodes to rapidly re-enter the bloodstream and CNS, resulting in major inflammatory states, with some comparing this phenomenon to the immune reconstitution inflammatory syndrome (IRIS) typically associated with the discontinuation of highly active antiretroviral therapy for patients with HIV [5,10]. Such a concept was corroborated by one experimental study demonstrating that discontinuation of fingolimod in mouse models with relapsing-remitting experimental autoimmune encephalitis resulted in the upregulation of S1P1 receptors in lymphocytes that were trapped in lymph nodes and that later allowed for travel to the CNS, shortly followed by severe relapse [17]. Another hypothesis suggests that fingolimod withdrawal results in astrocytic overexpression of S1P1, leading to the release of inflammatory cytokines and nitric oxide and resulting in a downstream inflammatory response, thus resulting in rebound episodes [5]. Other hypotheses focus on fingolimod’s effect on modulating T cell function. For example, one study showed the ratio of regulatory to pathogenic TH17 T cells rapidly returned to baseline after fingolimod discontinuation [18].

Various approaches to treating fingolimod rebound have been proposed, including the use of corticosteroids, plasma exchange, and B cell depletion with agents such as ocrelizumab [5]. Our patient had his first exacerbation two weeks after discontinuing fingolimod, received methylprednisolone 10 days later, received his first dose of ocrelizumab two weeks later, and then experienced another exacerbation five days later. Interestingly, there are two reported cases of patients with severe fingolimod rebound that were treated after more than eight weeks with the first dose of ocrelizumab and then experienced clinical worsening, perhaps due to ocrelizumab’s removal of regulatory B cells that have been hypothesized to have protective features or perhaps simply due to continued rebound activity unrelated to ocrelizumab [14]. Still, these patients improved or stabilized after continued treatment with ocrelizumab, reinforcing the benefit of its long-term administration [14].

Our patient’s fingolimod treatment was stopped due to his disseminated zoster infection, something that in itself has been associated with relapse in patients with MS. One study found the presence of VZV DNA in the blood and CSF of MS patients in relapse, with a decrease during disease remission, suggesting that VZV may play a role in MS relapse [19]. Another study found that a high percentage of MS patients had anti-VZV and that an abundance of VZV DNA had a significant correlation with MS disease [20], with implications that VZV may have a role in the pathogenesis of MS disease. Overall, the relationship between VZV and MS disease has still yet to be fully elucidated [19].

Our patient was incidentally found to be COVID-19-positive during his second MS relapse admission, though he was largely asymptomatic. Although infectious and inflammatory states are understood to contribute to possible MS exacerbations, the relationship between COVID-19 and MS is still being investigated. A recent study in the United Kingdom found that COVID-19 infection was associated with exacerbation of MS, finding that DMTs reduced the likelihood of developing new MS symptoms during the infection [21]. Another retrospective study also suggested that COVID-19 can trigger MS exacerbation [22]. There are several proposed mechanisms for how this may occur, including the hypothesis that COVID may invade the CNS through retrograde transport from the olfactory bulb, inducing glial inflammatory responses and BBB breakdown, or that COVID may stimulate autoreactive T cells against myelin through molecular mimicry [23].

Though the initiation of ocrelizumab and concomitant VZV and COVID-19 infection were considered in this patient as potential confounding factors, the temporal relationship between the patient's fingolimod discontinuation and MS exacerbations strongly suggests this phenomenon to be that of fingolimod rebound.

## Conclusions

Although there have been several studies on natalizumab rebound in patients with MS, fingolimod rebound is still not as well understood and largely debated. Here, we report the case of an older male patient with MS who was taken off his decade-long fingolimod treatment due to disseminated zoster infection and who subsequently experienced a rebound with two severe MS exacerbations within six weeks post-discontinuation. The phenomenon of fingolimod rebound has been reported to have a time course shorter than that of natalizumab-associated rebound, yet traditionally longer than was seen here. Fingolimod-associated rebound is a rare but important phenomenon for clinicians to consider, particularly when confounding influences may exist such as from infections like VZV and COVID-19 or even B cell depletion from biologic treatments such as ocrelizumab. Ultimately, this case highlights the importance of considering fingolimod rebound in patients who discontinue this medication and illustrates why proactive exit strategies from fingolimod and potential bridging strategies are essential, especially in older patients with infection risk. Furthermore, this case underscores the multifactorial nature of post-fingolimod disease activity and the importance of individualized transition strategies when managing high-efficacy DMT cessation.

## References

[1] Walton C, King R, Rechtman L (2020). Rising prevalence of multiple sclerosis worldwide: insights from the Atlas of MS, third edition. Mult Scler.

[2] McGinley MP, Goldschmidt CH, Rae-Grant AD (2021). Diagnosis and treatment of multiple sclerosis: a review. JAMA.

[3] Yaldizli O, Putzki N (2009). Natalizumab in the treatment of multiple sclerosis. Ther Adv Neurol Disord.

[4] Chaves C, Ganguly R, Dionne C, Camac A (2015). Relapse and rebound risks after natalizumab discontinuation in patients with multiple sclerosis. Neurology.

[5] Barry B, Erwin AA, Stevens J, Tornatore C (2019). Fingolimod rebound: a review of the clinical experience and management considerations. Neurol Ther.

[6] Vermersch P, Radue EW, Putzki N, Ritter S, Merschhemke M, Freedman MS (2017). A comparison of multiple sclerosis disease activity after discontinuation of fingolimod and placebo. Mult Scler J Exp Transl Clin.

[7] Maunula A, Atula S, Laakso SM, Tienari PJ (2024). Frequency and risk factors of rebound after fingolimod discontinuation - a retrospective study. Mult Scler Relat Disord.

[8] Goncuoglu C, Tuncer A, Bayraktar-Ekincioglu A (2021). Factors associated with fingolimod rebound: a single center real-life experience. Mult Scler Relat Disord.

[9] Barboza A, Gaitán MI, Alonso R (2022). Rebound activity after fingolimod cessation: a case-control study. Mult Scler Relat Disord.

[10] Berger B, Baumgartner A, Rauer S, Mader I, Luetzen N, Farenkopf U, Stich O (2015). Severe disease reactivation in four patients with relapsing-remitting multiple sclerosis after fingolimod cessation. J Neuroimmunol.

[11] Frau J, Sormani MP, Signori A (2018). Clinical activity after fingolimod cessation: disease reactivation or rebound?. Eur J Neurol.

[12] Hatcher SE, Waubant E, Nourbakhsh B, Crabtree-Hartman E, Graves JS (2016). Rebound syndrome in patients with multiple sclerosis after cessation of fingolimod treatment. JAMA Neurol.

[13] Lapucci C, Baroncini D, Cellerino M (2019). Different MRI patterns in MS worsening after stopping fingolimod. Neurol Neuroimmunol Neuroinflamm.

[14] Schmidt S, Schulten T (2019). Severe rebound after cessation of fingolimod treated with ocrelizumab with coincidental transient aggravation: report of two cases. Ther Adv Neurol Disord.

[15] Uygunoglu U, Tutuncu M, Altintas A, Saip S, Siva A (2018). Factors predictive of severe multiple sclerosis disease reactivation after fingolimod cessation. Neurologist.

[16] David OJ, Kovarik JM, Schmouder RL (2012). Clinical pharmacokinetics of fingolimod. Clin Pharmacokinet.

[17] Cavone L, Felici R, Lapucci A (2015). Dysregulation of sphingosine 1 phosphate receptor-1 (S1P1) signaling and regulatory lymphocyte-dependent immunosuppression in a model of post-fingolimod MS rebound. Brain Behav Immun.

[18] Ghadiri M, Fitz-Gerald L, Rezk A (2017). Reconstitution of the peripheral immune repertoire following withdrawal of fingolimod. Mult Scler.

[19] Sotelo J, Ordoñez G, Pineda B, Flores J (2014). The participation of varicella zoster virus in relapses of multiple sclerosis. Clin Neurol Neurosurg.

[20] Najafi S, Ghane M, Yousefzadeh-Chabok S, Amiri M (2016). The high prevalence of the varicella zoster virus in patients with relapsing-remitting multiple sclerosis: a case-control study in the north of Iran. Jundishapur J Microbiol.

[21] Garjani A, Middleton RM, Hunter R (2021). COVID-19 is associated with new symptoms of multiple sclerosis that are prevented by disease modifying therapies. Mult Scler Relat Disord.

[22] Barzegar M, Vaheb S, Mirmosayyeb O, Afshari-Safavi A, Nehzat N, Shaygannejad V (2021). Can coronavirus disease 2019 (COVID-19) trigger exacerbation of multiple sclerosis? A retrospective study. Mult Scler Relat Disord.

[23] MacDougall M, El-Hajj Sleiman J, Beauchemin P, Rangachari M (2022). SARS-CoV-2 and multiple sclerosis: potential for disease exacerbation. Front Immunol.

